# Study on Dynamic Crack Expansion and Size Effect of Back–Filling Concrete under Uniaxial Compression

**DOI:** 10.3390/ma16237503

**Published:** 2023-12-04

**Authors:** Xicai Gao, Huan Xia, Kai Fan, Leilei Yi, Jianhui Yin

**Affiliations:** 1 State Key Laboratory of Green and Low-Carbon Development of Tar-Rich Coal in Western China, Xi’an University of Science and Technology, Xi’an 710054, China; xiahuan1225@163.com; 2 Key Laboratory of Western Mine Exploitation and Hazard Prevention, Ministry of Education, Xi’an University of Science and Technology, Xi’an 710054, China; 3 School of Energy, Xi’an University of Science and Technology, Xi’an 710054, China; 4 Sichuan Coal Industry Group Huarong Co., Ltd., Panzhihua 617000, China; 5 Shaanxi Coal and Chemical Technology Institute Co., Ltd., Xi’an 710065, China

**Keywords:** gob-side entry retaining, back–filling concrete, YOLOv5, crack identification, size effect

## Abstract

With the continuous expansion of the application range of gob–side entry retaining technology, the depth, height, and advancing speed of coal seams also increase, which brings great problems to the stability control of surrounding rock structures of gob–side entry retaining. As one of the main bearing structures of the surrounding rock, the stability of the roadway–side support body is a key factor for the success of gob–side entry retaining. In order to study the deformation characteristics and instability mechanism of roadway-side support body, based on the roadway–side support materials of gob-side entry retaining, the dynamic expansion test of back–filling concrete cracks under uniaxial compression was carried out. The YOLOv5 algorithm was applied to establish the fine identification and quantitative characterization method of macroscopic cracks of the samples, and the dynamic expansion rule of roadway-side support body cracks and its dimensional effect were revealed by combining the fractal theory. The results show that the F1 value and average precision mean of the intelligent dynamic crack identification model reached 75% and 71%, respectively, the GIoU loss value tends to fit around 0.038, and the model reached the overall optimal solution. During the uniaxial compression process, micro cracks on the surface of the back–filling concrete first initiated at the end, and after reaching the yield stress, the macroscopic cracks developed significantly. Moreover, several secondary cracks expanded, pooled, and connected from the middle of the specimen to the two ends, inducing the overall instability of the specimen. The surface crack expansion rate, density, and fractal dimension all show stage change characteristics with the increase in stress, and the main crack expansion rate has obvious precursor characteristics. With the increase in the size, the decrease in crack density after back–filling concrete failures gradually decreases from 93.19% to 4.08%, the surface crack network develops from complex to simple, and the failure mode transits from tensile failure to shear failure. The above research results provide a basic experimental basis for design optimization and instability prediction of a roadway–side support body for engineering-scale applications.

## 1. Introduction

Gob–side entry retaining technology has significant advantages in alleviating the tension of mining replacements, reducing the loss of coal resources, and improving the efficiency of coal production; it has become an important development direction of green and efficient coal mining technology [1,2]. With the continuous expansion of the application scope of gob–side entry retaining technology, the increase in mining depth, mining height, and advancing speed of coal seams brings great problems to the control of surrounding rock structures, stress state, and stability control of roadway–side support [3]. Roadway–side support is one of the main methods for gob-side entry retaining in coal mines in China; reasonable and effective structural design of roadway–side support body has become a key factor for the success of gob–side entry retaining, and its stability is increasingly receiving more attention [4]. Therefore, from the perspective of controlling the surrounding rock structure of gob–side entry retaining, analyzing the effect of roadway–side support and balancing the pressure of surrounding rock is necessary. In addition, studying the performance degradation mechanism and size effect of back–filling concrete under high stress state is of great importance. It is of great significance for optimizing the size parameters of the deep roadway–side support body, and achieving long-term stability of the surrounding rock of the gob–side entry retaining. 

In recent years, our scholars have conducted a lot of work on the stress state, deformation characteristics, and width optimization design of roadway–side support in gob-side entry retaining [5]. Feng Guorui et al. [6] calculated the working resistance of roadway–side back–filling body by establishing a superimposed laminate model, and analyzed the stress distribution and deformation characteristics of roadway–side back–filling body. Han Changliang et al. [7] revealed the collapse characteristics and superimposed disturbance mechanism of the overlying strata of the gob–side entry retaining through physical simulation and theoretical analysis, and proposed a method for calculating the load on the back–filling wall. Chen et al. [8] believed that the high stress of the roadway–side back–filling body during the breaking movement of the overlying strata is the root cause of the initiation, development, and penetration of the surface cracks of the roadway–side back–filling body and the deterioration of its own bearing capacity. Zhang Jixiong, He Fengzhen, and Feng Chao et al. [9,10,11] calculated the reasonable width of the roadway–side back–filling body, and analyzed the maintenance effect of gob–side entry retaining with different widths based on the field engineering geological conditions, which laid a solid foundation for the stability control of the surrounding rock and the popularization and application of the technology. However, with the continuous increase in coal seam mining depth, deep gob–side entry retaining will face more complex stress environments and mining technical conditions; the roadway–side support body deserves further study in terms of back–filling material, instability mechanism, and stability control.

The expansion and penetration of the roadway–side filling body cracks is the key factor for the instability of its body under the balanced pressure of the overlying strata of mining face [12,13,14], proactively identifying and analyzing the dynamic crack expansion law of the road–side back–filling body in advance is essential for assessing the overall stability of filling body [15,16]. In terms of intelligent identification of dynamic expansion of rock mass cracks, deep learning methods are increasingly used in laboratory and field real–time monitoring. Liu Yu, Wu Peirong, and Ye Guanting et al. [17,18,19] proposed an improved single–stage target detection network model for the accurate identification of concrete cracks at different scales. Jiang yongqing et al. [20] realized the detection and classification of concrete surface damage through deep separable convolution, inverse residual network, and linear bottleneck structure optimization target detection algorithms. Cui Xiaoning et al. [21] achieved an accurate identification of concrete erosion damage based on the YOLO algorithm model. Song Ee Park et al. [22] proposed a target detection method by combining deep learning and structured light technology that was capable of real-time and high–precision detection and quantification of structural surface cracks. This provided a new approach for advanced identification of coal and rock mass fractures and the capture of instability precursor information.

Therefore, the author considers the bearing stress state of the roadway–side support body of gob–side entry retaining by conducting dynamic crack extension tests on roadside filling concrete materials. The high–speed camera was used to record the deformation and failure process of the sample in real time, and the expansion and evolution characteristics of the main cracks were quantitatively analyzed. Based on the YOLOv5 algorithm, a fine identification and characterization method of macroscopic crack expansion was established and combined with fractal theory. The mechanical behavior, crack dynamic expansion law, and size effect of back–filling concrete under the action of roof equilibrium pressure are analyzed. It provides the basic test basis for the design optimization and instability prediction of gob–side entry retaining back–filling bodies for engineering–scale applications.

## 2. Mechanical Test Design

### 2.1. Sample Preparation

**Figure 1:**
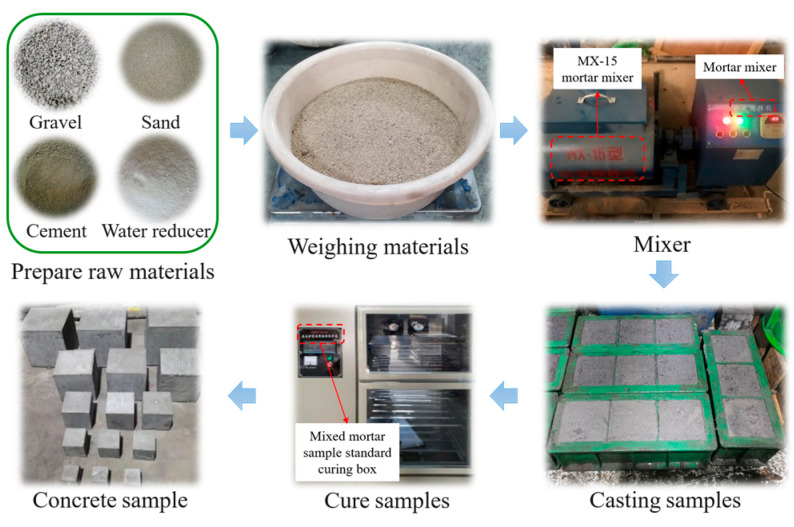
Sample preparation procedures.

Procedures were based on the ratio of back–filling concrete materials along the gob-side entry retaining in 3307 working faces of a mine. Cement (PO 42.5), gravel (particle size grading 5–16 mm), river sand (modulus in 2.6–3.0), water reducing agent, and water according to the proportion of 0.56:0.85:1:0.0013:0.32 were mixed and stirred for 3 min. Pour the stirred slurry into the mold and vibrate it thoroughly for 30 s. Let it stand for 24 h before demolding. Place the sample in a standard curing box, set the temperature at 20 ± 2 °C, relative humidity of 90%, cure 28 d after the preparation of back–filling concrete samples [23]. The preparation process is shown in Figure 1. A total of 5 groups of size samples were prepared for this test, 3 samples in each group, and the sample sizes and numbers are shown in Table 1.

### 2.2. Test Equipment and Methods

**Figure 2:**
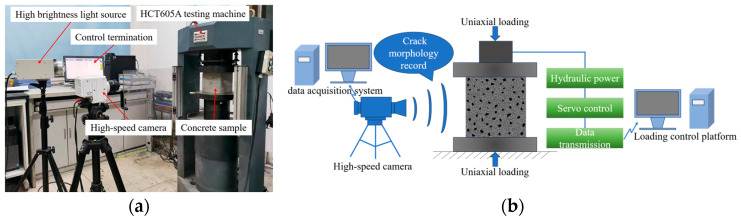
Integrated test system. (**a**) Sample loading and recording system. (**b**) Test principle.

The sample loading and recording systems are shown in Figure 2a, and the testing principle is shown in Figure 2b. The uni–axial compression test was carried out using a microcomputer–controlled electro–hydraulic servo pressure testing machine (Shenzhen Wance Testing Equipment Co., Ltd., Shenzhen, China), setting a stress control mode, and a loading rate of 0.5 MPa/s. The development and evolution characteristics of surface cracks were recorded using a Phantom VE0710L high-speed camera (York Technologies Limited, Hong Kong, China) during the loading process in real-time, with a resolution of 512 × 256, shooting frame rate 400FPS, exposure time 30 μs, the exposure index was 20,000.

## 3. Intelligent Identification Method of Back–Filling Concrete Cracks Based on YOLOv5 Algorithm 

### 3.1. YOLOv5 Detection Algorithm

The image of crack expansion and evolution on the surface of the sample were obtained during the loading process of back–filling the concrete, and an intelligent detection model based on the YOLOv5 algorithm was built to realize the intelligent identification of dynamic cracks of back–filling concrete samples under uniaxial compression.

**Figure 3:**
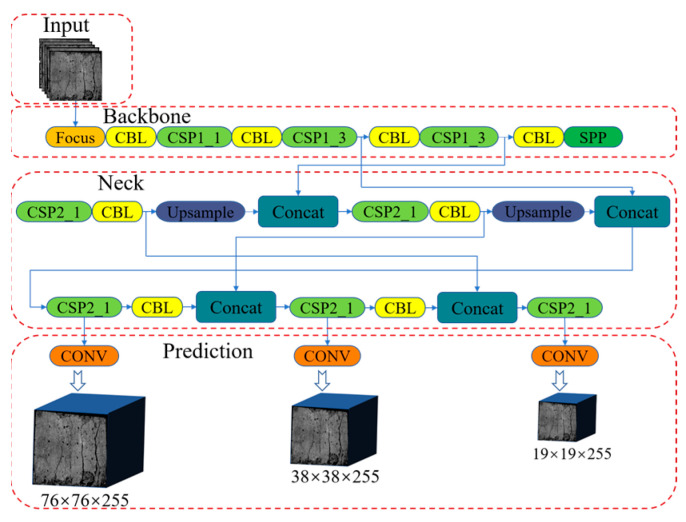
YOLOv5 network structure.

The network structure of the YOLOv5 detection algorithm is shown in Figure 3. The YOLOv5 algorithm based on Pytorch has advantages such as fast detection speed, high accuracy, and lightweight model; its network structure is mainly composed of input, backbone, neck, and prediction. Input was mainly composed of Mosaic data enhancement, adaptive anchor frame calculations, and adaptive crack expansion image scaling. Backbone mainly includes focus structure, CSP (cross stage partial) structure, CBL structure, and SPP (spatial pyramid pooling) structure. The focus structure sliced the input crack expansion image to obtain a double sampling feature map with complete information [24]. The CSP structure divided the input crack expansion image into two parts and a convolution operation was performed on them, respectively, and then the results of the two parts were spliced, which can increase the depth of the network structure, maintain the computational efficiency of the network structure, and improve the model learning ability [25]. CBL is a standard convolutional structure consisting of a common convolution layer (Conv), a batch normalization layer (BN), and an activation function layer (LeakyReLU). The SPP structure increased the receptive field range and feature expression ability of the model by maximum pooling after multiple convolutions of the feature layer [26]. The neck section is based on upsampling and Concat modules, adopts the structure of FPN + PAN, which conveyed high-level semantic information from top to bottom, and transmitted positioning features from bottom to top, complementing each other, significantly enhancing the network structure feature fusion ability [27]. In the prediction stage, GIOU_LOSS was used as the loss function of the bounding box, which greatly improved the accuracy of the bounding box. The NMS non-maximum suppression was used to screen the optimal bounding box at each target position and eliminate the redundant bounding box. Simultaneously, the Conv structure is used to downsample the input image and extract target features.

### 3.2. Detection Method Steps

**Figure 4:**
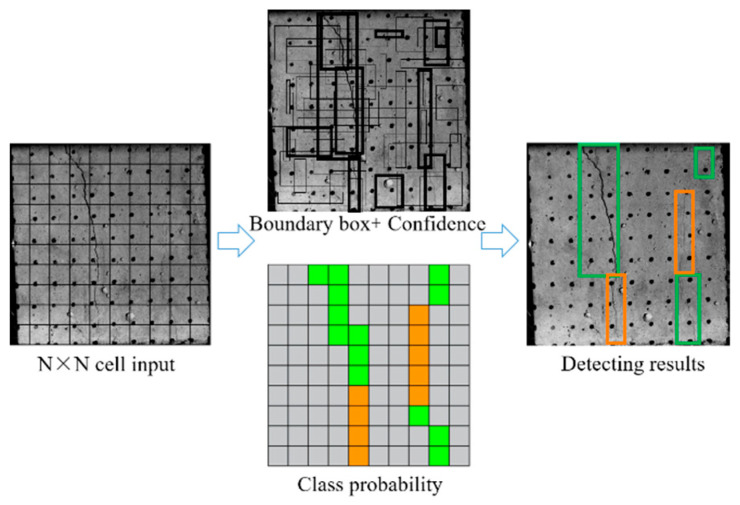
Dynamic crack detection and identification process of back–filling concrete samples.

The dynamic surface crack detection and recognition process of the sample is shown in Figure 4.

(1) Dividing the crack image into N × N grid units for image feature extraction, each cell generates a bounding box for different targets, with each bounding box containing the center point position (x, y), bounding box width and height (w, h), and conf (confidence). conf is a measure of the accuracy of the model’s prediction of the target box.
  (1) $$conf=Pr\left(object\right)\times IoU\left(pred,truth\right)\times Pr\left(class\right)$$
  where *conf* is divided into three part: *Pr* (*object*) represents the probability of the existence of the target. If there is no crack in the prediction box, *Pr* (*object*) = 0, otherwise it is 1; *IoU* (*pred, truth*) represents the intersection and union ratio of the prediction box and the real box; *Pr* (*class*) represents the probability that the prediction box belongs to each category.
(2) Extracting features from the normalized data set using a feature extraction network.
(3) Setting prediction box, calculating the coordinates of the center point for the different detection targets.
(4) Calculating the position of the target center point and the width and height of the prediction box based on the predicted coordinate offset value.
(5) Output crack detection results.

### 3.3. Construction of Crack Expansion Image Data Set

**Figure 5:**
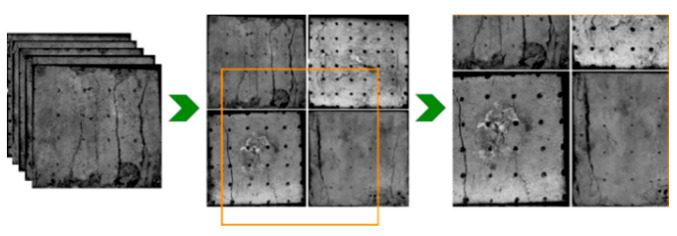
Principles of Mosaic data enhancement technology.

Creation of deep learning data sets from crack extension images of sample surfaces recorded by a high–speed camera during the loading process. A total of 2000 sample images of specimen surface cracks were selected for the loading process of 5 groups of size specimens; a total of 1500 labeled sample images were used as the training set and 500 unlabeled sample images were used as the test set. The open source software Labelimg (https://github.com/tzutalin/labellmg) was used to mark the cracks in 1500 sample images. The surface cracks on the image were marked as tensile cracks (TFs) and compression shear cracks (CSFs), respectively. At the same time, the Mosaic data enhancement technology was used to expand the data set [28]. The multiple crack expansion images were selected randomly and scaled, segmented, and then spliced into one image, which enriched the sample image data set to make the detection of small cracks more accurate, and improved the universality and robustness of the YOLOv5 network structure. The principles of data enhancement technology are shown in Figure 5. 

### 3.4. Analysis of Detection Results

**Figure 6:**
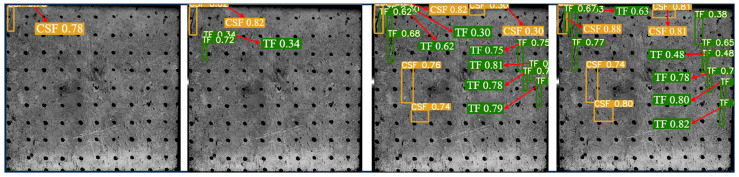
Dynamic crack test results of back–filling concrete samples. Note: Two colors of the prediction box (green and orange) correspond to two different types of cracks: TF and CSF. The value in the prediction box is confidence. Table format for output results can be found in Table S1.

The YOLOv5 algorithm model was built using Python 3.8 and Pytorch 2.0.0 versions. The input image size was 640 × 640 Pixels, the weight attenuation factor was 0.001, the momentum coefficient of the model was 0.98, the learning rate was 0.1, and the iterative training was 100 rounds. After the training was completed, the test set image was input into the trained YOLOv5 model, and the target prediction box and confidence were output and the surface cracks detection results of the sample were shown in Figure 6. The YOLOv5 algorithm identified and marked all the surface cracks on the sample. The classification was accurate, the recognition accuracy was high, and the average confidence was 0.7.

### 3.5. Algorithm Evaluation

#### 3.5.1. Model Recognition Accuracy Measurement Indicators

(1) F1 value (*F-Measure*)

In order to evaluate the model more comprehensively, a comprehensive index *F-Measure* was used combining *P* (precision rate) and *R* (recall rate) as the evaluation index, and the calculation formula was shown in Equation (2).

(2) $$F-Measure=\frac{2PR}{P+R}$$

**Figure 7:**
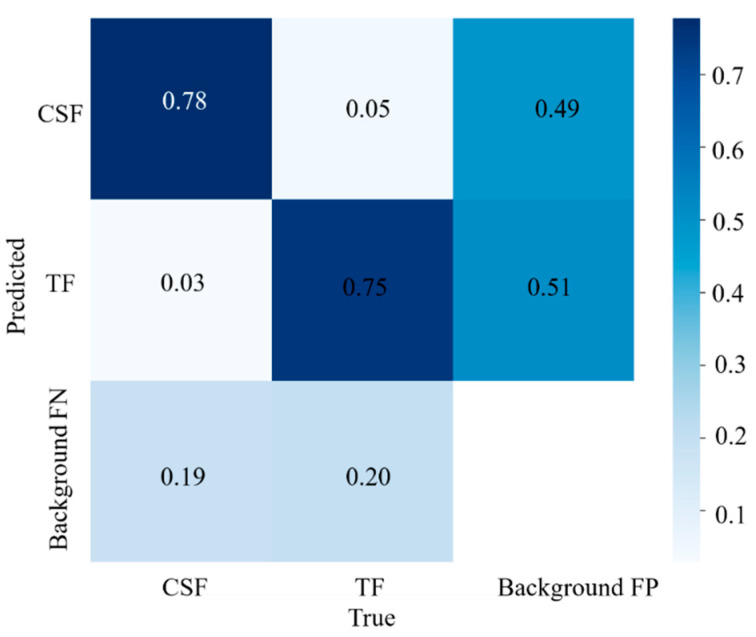
Identification of the model confusion matrix.

The *F*–*Measure* depended on the calculation of the confusion matrix, *P* (precision rate) and the *R* (recall rate), where the confusion matrix was a summary of the prediction results for crack classification problems. The confusion matrix obtained from YOLOv5 model training is shown in Figure 7.

The results of YOLOv5 model detection are as follows:

*TP*: The model predicts CSF and the label value is also CSF, indicating correct detection results;

*FN*: The model predicts TF, the labeled value is CSF, and the detection result is incorrect;

*FP*: The model predicts CSF, the label value is TF, and the detection result is incorrect;

*TN*: The model predicts TF and the label value is also TF, indicating correct detection results.

The *P* (precision rate) is the proportion of TF and CSF in all predictions, which measures the accuracy of the prediction; the *R* (recall rate) represents the accuracy rate of each category, which measures whether the detection is comprehensive. The calculation formula is shown in Equations (3) and (4).

(3) $$P=\frac{TP}{FP+TP}$$

(4) $$R=\frac{TP}{FN+TP}$$

(2) Average precision mean (*mAp@0.5*)

The average precision mean (*mAp@0.5*) was used to evaluation model, *mAp* can measure the performance of various label predictions. The higher the *mAp*, the better the performance.

(3) Generalized loss function (*GIoU*)

The generalized loss function evaluation model was adopted. The smaller the *GIoU* loss value, the better the detection convergence effect. The specific calculation formula is shown in Formulas (5)–(7).

(5) $$IoU=\frac{\left|A\cap B\right|}{\left|A\cup B\right|}$$

(6) $$GIoU=IoU-\frac{\left|C/(A\cup B)\right|}{\left|C\right|}$$

(7) $$GIoU_{loss}=1-GIoU$$

where *GIoU_loss_* is the loss value; *A* and *B* are the area of the target box and the prediction box, respectively; *C* is the minimum closure area of two boxes.

#### 3.5.2. Evaluation Results Analysis

**Figure 8:**
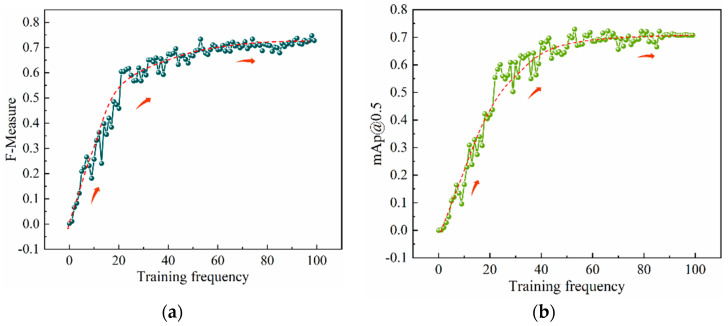
Model training curves. (**a**) *F*–*Measure* curve. (**b**) *mAp@0.5* curve.

The training curve of the intelligent recognition algorithm model is shown in Figure 8. With the increase in the number of iterations training, the F1 value (*F*–*Measure*) and the average precision mean (*mAp@0.5*) gradually increased. When the iteration was close to 100 times, the F1 value and the average precision mean curve reached an equilibrium. After balancing, the F1 value and the average precision mean reached more than 70%, the F1 value was 75%, and the average precision mean was 71%. It shows that the constructed model achieved a better training effect.

**Figure 9:**
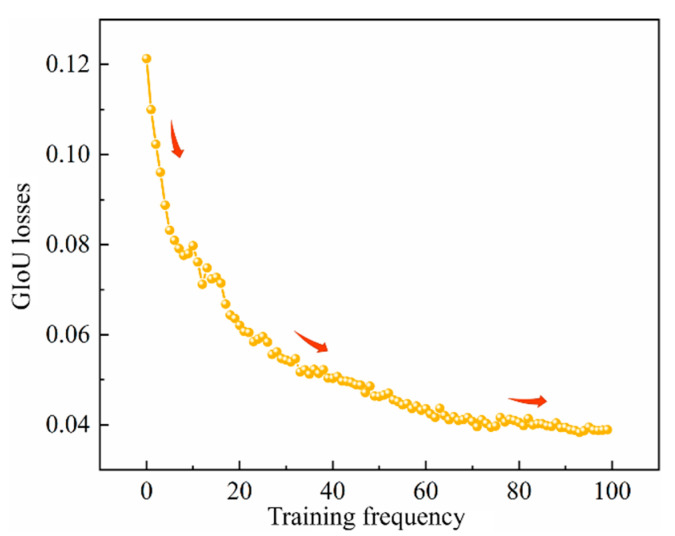
*GIoU_loss_* function curves.

The curve of *GIoU* loss function with the number of iterative training times is shown in Figure 9. The *GIoU* loss rate curve shows a downward trend. The initial loss value of the model is about 0.124, the *GIoU* loss value reached 0.038 after 100 iterative training, and the model tended towards a stable state. It showed that after 100 iterative training, the YOLOv5 model achieved the overall optimal solution and the detection convergence effect was better.

## 4. Cracks Dynamic Expansion Law of Back–Filling Concrete under Uniaxial Compression

### 4.1. Crack Expansion and Collection

**Figure 10:**
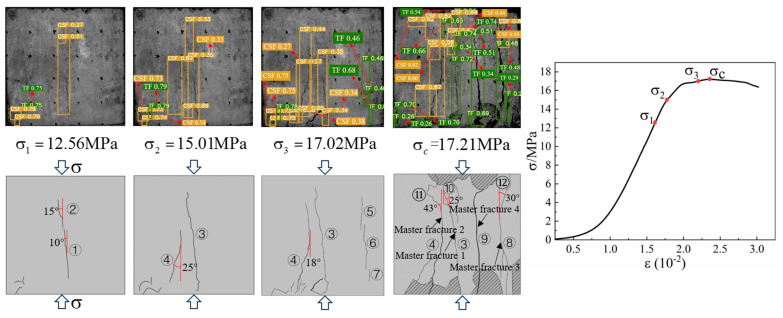
Macro crack expansion and evolution characteristics of back–filling concrete.

Based on the YOLOv5 crack identification algorithm, the surface crack expansion and evolution characteristics of the sample were obtained as shown in Figure 10. During the uniaxial compression process, micro cracks on the surface of the back–filling concrete sample first initiated at the end, and multiple secondary cracks developed in the middle of the sample and expanded and converged towards both ends. After reaching the yield stress, the cracks entered an unstable development stage, and the secondary cracks gradually connected to form one or more master cracks until instability and failure occurred.

Due to space limitations, sample Sj200 was taken as an example. At the initial stage of loading, the original cracks inside the sample were compacted and closed during the initial loading stage, the stress–strain curve showed an upward concave shape, and there were no cracks developed on the surface of the sample. When the stress was loaded to 9.97 MPa, the microcracks inside the sample gradually developed and expanded, and secondary cracks sprouted in the lower left corner of the sample under the influence of the end effect. When the stress was loaded to 12.56 MPa, cracks ① and ② simultaneously initiated in the middle of the sample and continuously expanded towards both ends, with angles of 10°and 15° with the direction of the loading stress, respectively. When the loading stress was 15.01 MPa, cracks ① and ② intersected to form cracks ③ with an angle of 15° to the direction of loading stress. At the same time, cracks ④ initiated and developed from the lower left end of the sample upwards, with an angle of 25° to the direction of loading stress. When the stress was loaded to 17.02 MPa, the stress–strain curve deviated from the straight line, and cracks ③ and ④ continue to expand upward. In addition, cracks ⑤, ⑥, and ⑦ appeared on the right side of the sample, which were paralleled to the direction of loading stress. When the loading stress reached 17.21 MPa, the cracks ⑤, ⑥, and ⑦ on the right side of the sample were connected to each other to form crack ⑧. The cracks ⑩, ⑪, and ⑫ at the upper end of the sample expanded downward, and were connected with cracks ③, ④, and ⑧, respectively, to form penetrating master cracks 1, 2 and 3. At the same time, crack ⑨ expanded rapidly from the lower end of the sample in the direction of parallel principal stress, and connected with the crack zone at the upper end to form penetrating master crack 4, the overall instability rupture of the sample occurred due to the penetration of the main cracks 1, 2, 3, and 4. It can be seen that the master crack 4 was approximately parallel to the direction of loading stress, and that it was a tension crack. The angles between the master cracks 1, 2, and 3 and the direction of loading stress are 25°, 43°, and 30°, respectively, all of which were compression shear cracks. When the sample was destroyed, the master crack was mainly compression-shear crack.

### 4.2. Fractal Characteristics of Crack Expansion and Evolution

The crack expansion and evolution law of the surface of the back–filling concrete sample was indirectly reflected through calculating the fractal dimension of the surface of the sample under different loading stress levels [29,30,31]. The calculation of fractal dimension based on box counting method is shown in Formula (8):

(8) logN(d)=logA−Dlogd

where *A* is constant; *d* is the grid side length, mm; *N*(*d*) is the number of grids covering the crack zone; and *D* is the fractal dimension.

**Figure 11:**
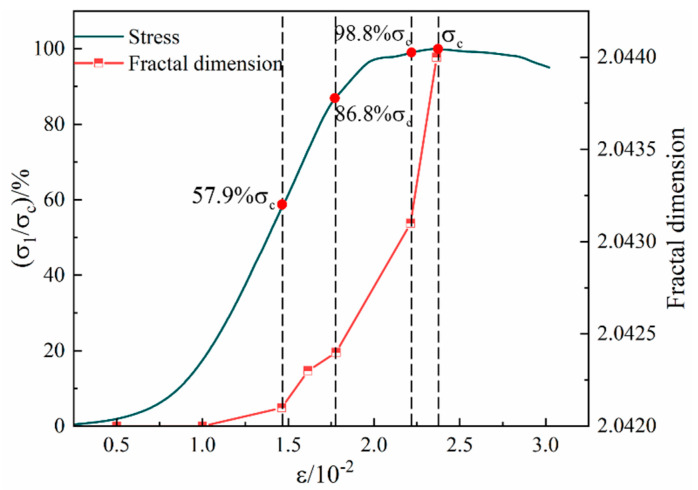
The relationship curve between fractal dimension and stress level of back–filling concrete.

The relationship between the fractal dimension and the stress level during the loading process of the Sj200 sample is shown in Figure 11. During the uniaxial compression process, the fractal dimension of the surface cracks of the sample was basically consistent with the evolution characteristics of the stress–strain curve, showing a phased change characteristic of slowly increasing first and then suddenly rising. As the loading progresses, the fractal dimension of the surface cracks of the sample shows a slow increasing trend with increasing stress, and the complexity of the surface crack network of the sample gradually increases. When the loading stress value reached 57.9% σ_c_, there was a secondary crack initiation on the sample surface, and the fractal dimension is 2.0421. When the stress was loaded to 86.8% σ_c_, the fractal dimension increased to 2.0424. At this time, the sample reached the yield stress limit, and the crack development entered the unstable development stage. As the stress level continued to increase, a large number of cracks inside the sample expanded and converged to form a macroscopic master crack, and a large number of secondary cracks developed around the master crack. At this time, the growth trend of fractal dimension changed from a slow increase to a sudden increase, and the complexity of the crack network on the surface of the sample increased. When the stress was loaded to 98.8% σ_c_, the crack further expanded to form multiple penetrating master cracks. The fractal dimension was 2.0431, and the fractal dimension growth rate increased again. The fractal dimension suddenly increased to the maximum value of 2.0440 at the peak stress.

### 4.3. Characteristics of Master Crack Expansion Rate

**Figure 12:**
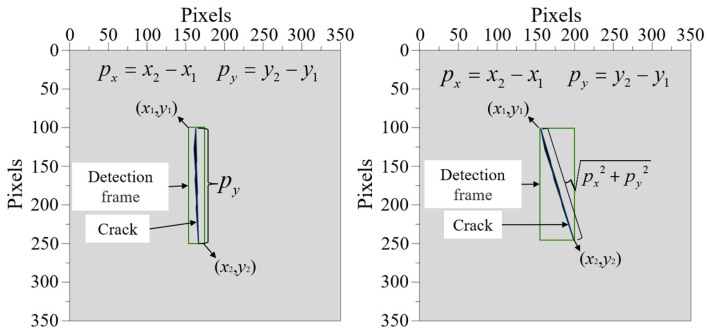
Calculation diagram of crack propagation length.

During the loading process of the sample, the high-speed camera was used to capture the crack expansion process at a frame rate of 400 FPS. The coordinates of the upper left and lower right corners of the detection frame for the real–time expansion position of cracks were obtained through the YOLOv5 algorithm model. If the crack expansion direction was approximately parallel to the loading direction, the ordinate of the detection frame were subtracted, and the difference values can be substituted into Equation (9) to obtain the crack expansion length ∆*l*. If the crack expansion direction and the loading direction were at a certain angle, the horizontal and vertical coordinates of the detection frame were processed by their difference, the obtained horizontal and vertical coordinates were substituted into Equation (10), respectively, and the crack expansion length ∆*l* can be also obtained. The calculation of crack expansion length is shown in Figure 12. In addition, the crack expansion rate *v* (mm/s) is the ratio of the crack expansion length Δ*l* to the time *t* required for the crack expansion.

(9) $$\Delta l=\frac{d\times p_{y}}{p}$$

(10) $$\Delta l=\frac{d\times \sqrt{p_{x}{}^{2}+p_{y}{}^{2}}}{p}$$

where *d* is the sample size, mm; *P_x_* is the difference in the horizontal axis of the detection boxes, pixels; *P_y_* is the difference in the vertical coordinates of the detection boxes, pixels; *P* is the pixel height of the sample, pixels.

**Figure 13:**
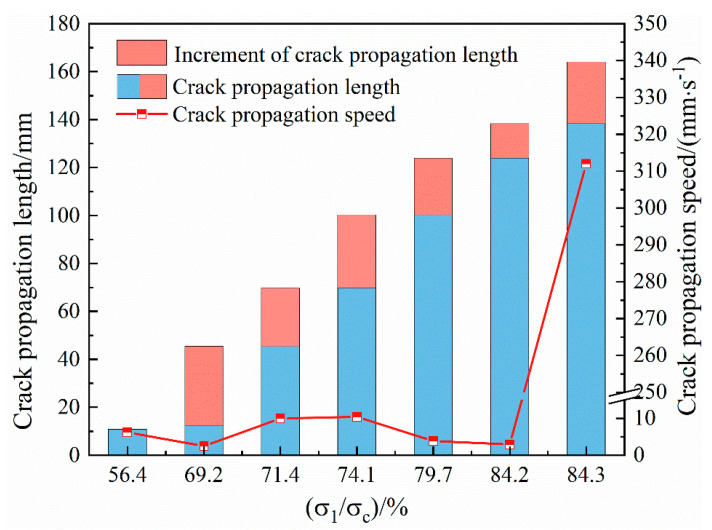
The relationship curve between the crack propagation speed and the stress level of the back–filling concrete.

The relationship curve between the master crack expansion rate and the stress level of the Sj200 sample is shown in Figure 13. During the uniaxial loading process of back–filling concrete, the propagation rate of the master crack showed a phased change characteristic of ‘slow decrease—minor fluctuations—sudden increase’ with the increase in stress. After loading to the yield stress, the master crack expansion rate of the sample suddenly increased, forming one or more through cracks in a short period of time and the brittle instability failure of the sample happened. The sudden change in crack expansion rate can be used as a key indicator to determine the precursor information of back–filling concrete failure.

When the stress was loaded to 56.4% σ_c_, the secondary cracks converged to form the master crack 1, the total length of crack expansion was 10.85 mm, and the corresponding initial crack expansion rate was 6.17 mm/s. With the increase in stress, the crack was in a stable expansion stage. When the stress was loaded to 69.2% σ_c_, the crack expansion length was 45.45 mm, and the expansion rate was reduced to 2.46 mm/s. During the propagation process, the expansion rate of the master crack showed a slow decrease or a small fluctuation (2.46~10.40 mm/s) because of the larger aggregates or particles with strong cementation. When the stress was loaded to 84.3% σ_c_, the crack entered the unstable expansion stage, with the master crack 1 expanded to 164.05 mm in a short time, and the expansion rate suddenly increased to the maximum value of 312 mm/s.

### 4.4. The Influence of Size Change on the Dynamic Expansion Law of Cracks

The crack density *M* was defined as the ratio of the number of surface cracks W on the back–filling concrete sample to its surface area S, and it can be quantitatively analyzed the crack expansion law of the back–filling concrete under uniaxial loading conditions.

**Figure 14:**
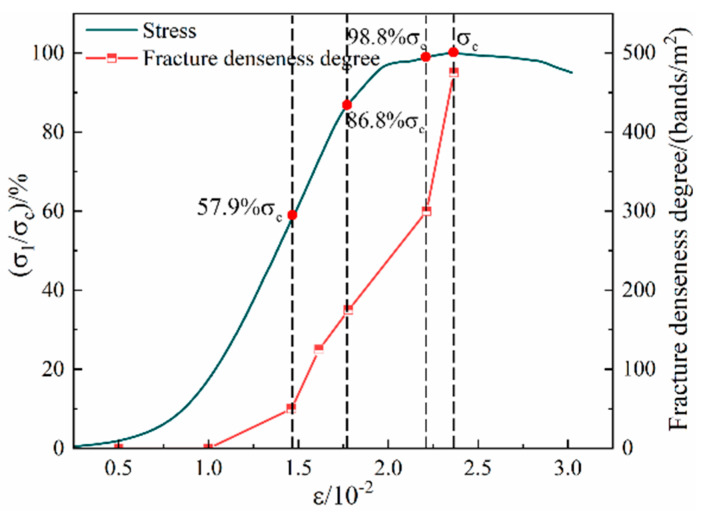
The relationship curve between crack density and stress level of back–filling concrete.

The relationship curve between crack density and stress level of Sj200 sample is shown in Figure 14. Under uniaxial loading, the number of surface cracks increased with the increase in stress level, and the increase in crack density amplification showed the characteristics of stage change. When the stress of the sample was loaded to 57.9% σ_c_, the internal small–scale cracks began to develop, expand, and connect with each other. The macroscopic performance was the initial crack initiation, and the crack density increased from 0 to 50 bands/m^2^. As the loading continues, the internal micro cracks of the sample develop steadily, and secondary cracks continued to sprout on the surface, promoting a slow increase in crack density. When the stress was loaded to 86.8% σ_c_, the crack density increased to 175 bands/m^2^. The sample reached the yield stress limit, forming multiple macro master cracks and accompanying a large number of secondary cracks. When the stress was loaded to 98.8% σ_c_, the crack density suddenly increased, and the crack density increased to 300 bands/m^2^. When the peak stress σ_c_ was reached, the crack density increased to a maximum of 475 bands/m^2^.

**Figure 15:**
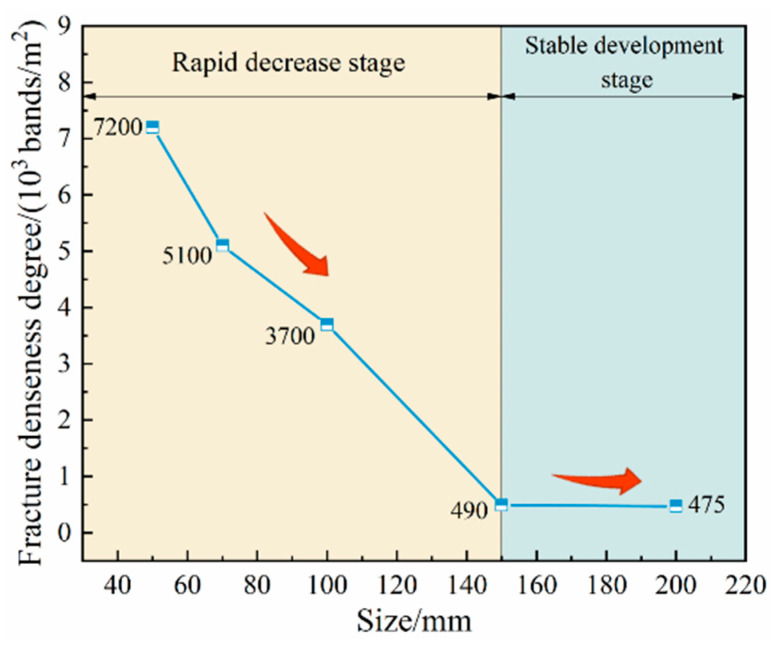
Crack density with size change curve.

Due to the different sizes of the back–filling concrete samples, there were some differences in the crack density after the failure of the samples. The curve of crack density with size was shown in Figure 15. When the size increased from 50 mm to 150 mm, the difference of crack density was 6710 bands/m^2^, and the decrease was 93.19%. When the size increased from 150 mm to 200 mm, the difference of crack density was 20 bands/m^2^ and the decrease was only 4.08%. It can be seen that under uniaxial compression, the crack density of the back–filling concrete sample showed the characteristics of first decline before stabilization with the increase in the size.

Typical failure characteristics of samples with different sizes are shown in Table 2. When the size of the back–filling concrete sample was small, the master crack after sample failure was mainly tensile crack, and the failure mode was tensile failure. After the sample exceeded 150 mm, the development of secondary cracks was less, and the development of through compression shear cracks was obvious. The failure mode gradually transitioned from tensile failure to shear failure. When the Sj50 and Sj70 samples were damaged, the tensile master cracks were link up and accompanied by a large number of secondary cracks. When the sample of Sj100 was damaged, the left master cracks were parallel to the principal stress direction, the angle between the right master cracks and the principal stress direction was about 30°. At the same time, some secondary crack were developed at the end, the failure mode was tensile and shear compound destruction. When the samples of Sj150 and Sj200 were destroyed, the master cracks of compression–shear were penetrated. In addition, the master cracks were roughly in the form of X–shaped conjugate inclined plane shear, the number and density of macro cracks were obviously reduced, and spalling bodies appeared on the local surface; the failure mode was mainly shear failure. In the process of uniaxial compression failure, the lateral deformation of the sample was large and the volume expansion phenomenon was significant.

## 5. Conclusions

(1) The dynamic expansion test of roadway–side back–filling concrete cracks under uniaxial compression was carried out. An intelligent identification algorithm for dynamic cracks of back–filling concrete samples was established by using a convolutional neural network YOLOv5 based on the real–time recording images of the surface cracks of the samples. The evaluation results show that the F1 values of the evaluation index and the average precision mean of the identification model reached 75% and 71%, respectively, and the GIoU loss value was stable at 0.038; the YOLOv5 model achieved the overall optimal solution and the detection convergence effect was good, which can intelligently and accurately identify dynamic cracks.
(2) The micro cracks on the surface of the back–filling concrete first initiated at the end during the uniaxial compression process. As the loading progressed, multiple secondary cracks developed in the middle of the sample, and expanded and converged to both ends. After reaching the yield stress, the surface cracks of the sample expanded unstably, and the secondary cracks gradually penetrated to form one or more master cracks until they fail. The surface cracks of back–filling concrete samples increased with the increase in stress level, and the increase in crack density and fractal dimensions showed the characteristics of stage change. The expansion rate of the master crack showed the stage characteristics of a “slow decrease—minor fluctuations—sudden increase”; with the increase in stress, a macroscopic through master crack of time can be formed in a short period. The precursory characteristics of crack development of back–filling concrete were significant.
(3) The size effects of the dynamic crack expansion law and failure mode of back–filling concrete were obvious. After the failure of the back–filling concrete, the crack density rapidly decreased and then steadily developed with the increase in the size. When the size was small, the crack network on the surface of the back–filling concrete tended to be complex, and the failure mode of the sample was mainly tensile failure. As the size increased, the crack network developed from complex to simple and the failure mode gradually transitioned from tensile failure to shear failure. At the same time, the research results can provide some reference for the mechanical properties experiment of back–filling concrete and the optimization design of field size.

## 6. Future Prospect

This article prepared samples of different sizes of back–filling concrete and conducted dynamic crack extension experiments on back–filling concrete under uniaxial compression. The YOLOv5 algorithm was used and was combined with fractal theory to reveal the dynamic crack expansion rule and size effect of the roadway–side support body. In the future, the discrete element numerical simulation method can be used to study the dynamic crack expansion rule and size effect of larger size filling concrete. The above research results provide a basic experimental basis for the design optimization and instability prediction of a roadway–side support body for engineering–scale applications.

## Figures and Tables

**Table 1:** Sample size and number.

**Sample size/mm**	50 × 50 × 50	70 × 70 × 70	100 × 100 × 100	150 × 150 × 150	200 × 200 × 200
**Sample number**	Sj50	Sj70	Sj100	Sj150	Sj200

**Table 2:** Failure characteristics of back–filling concrete samples.

Sample Number	Sj50	Sj70	Sj100	Sj150	Sj200
Crack identification	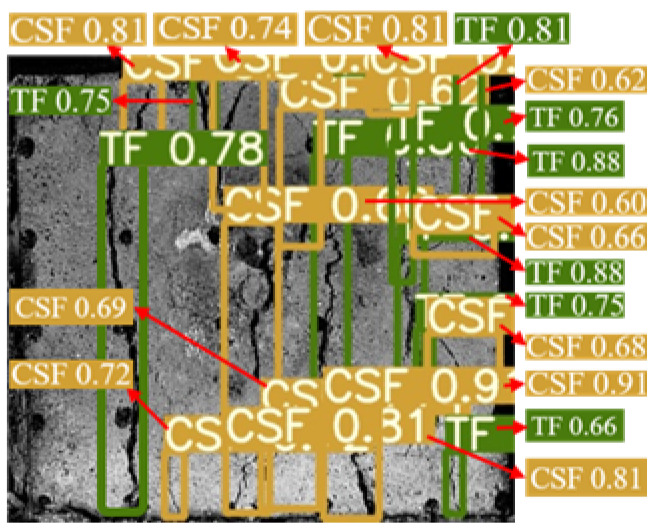	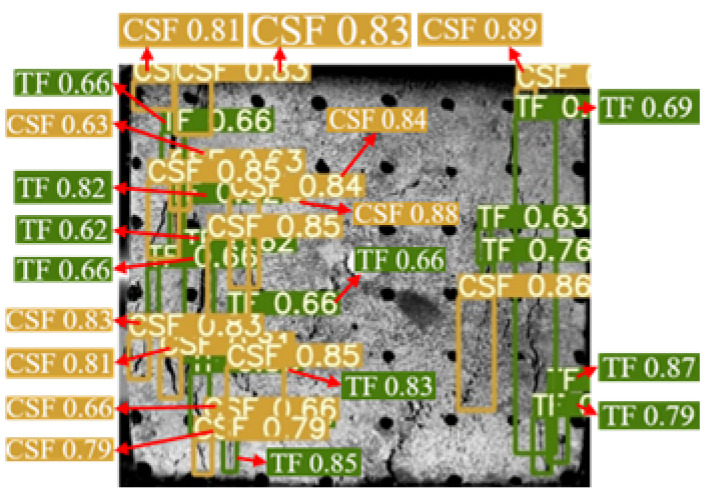	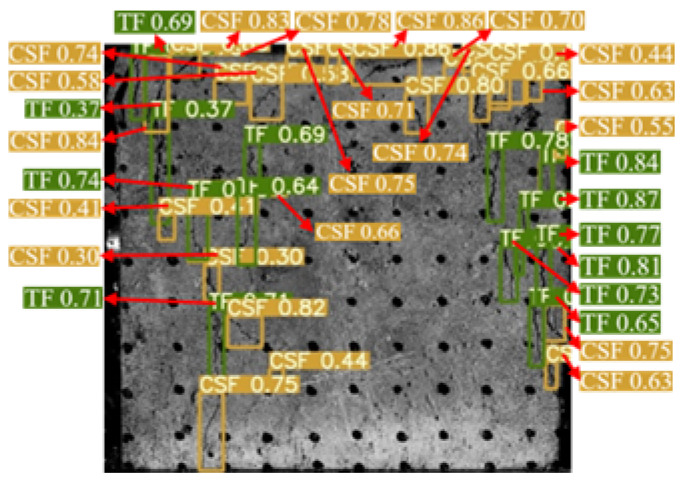	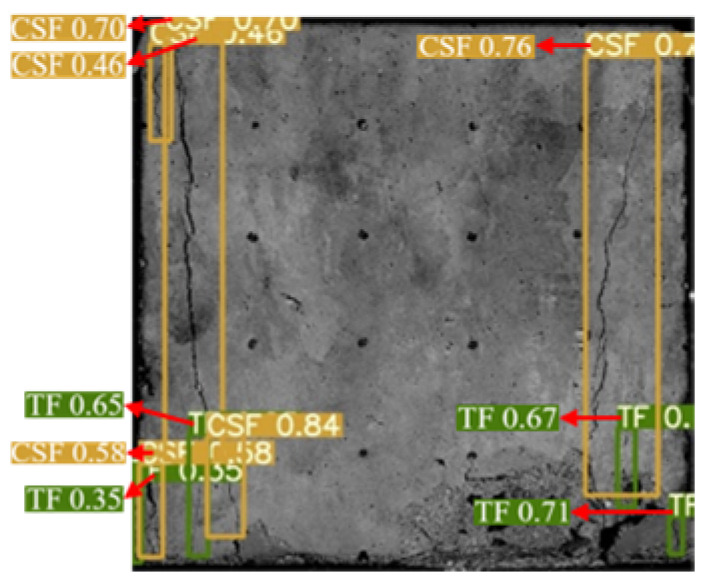	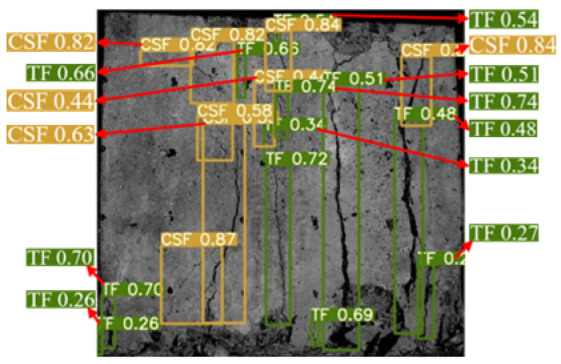
ketch	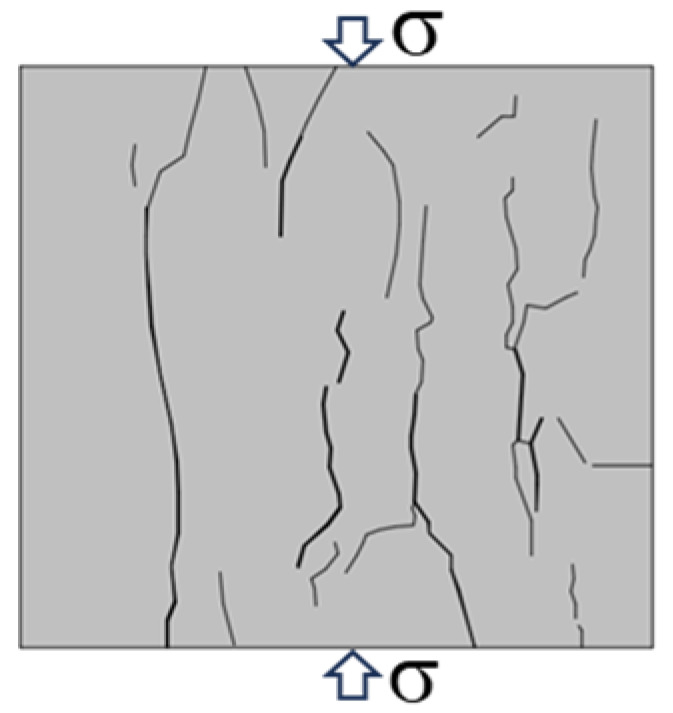	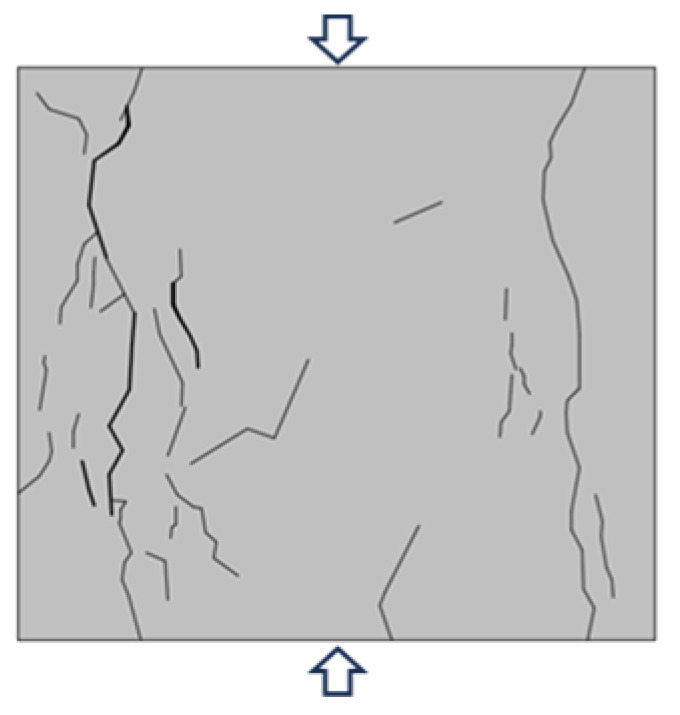	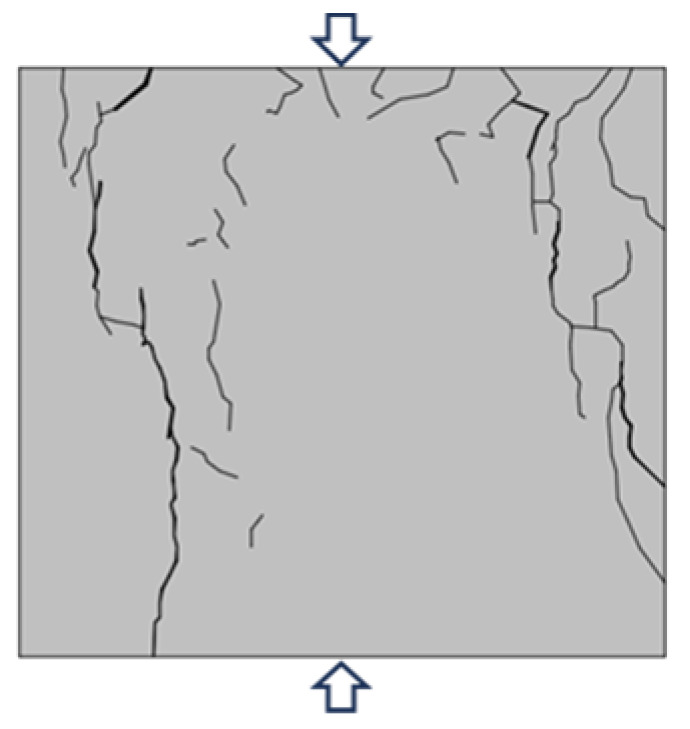	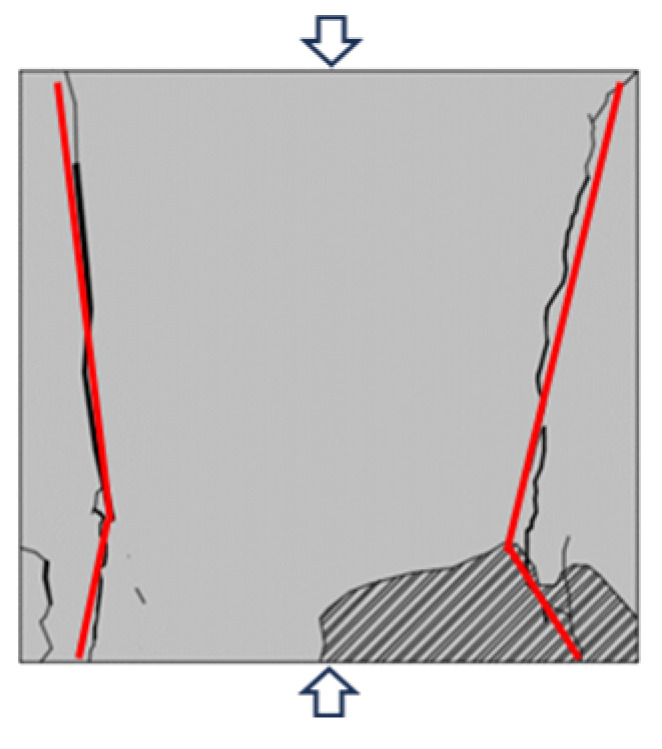	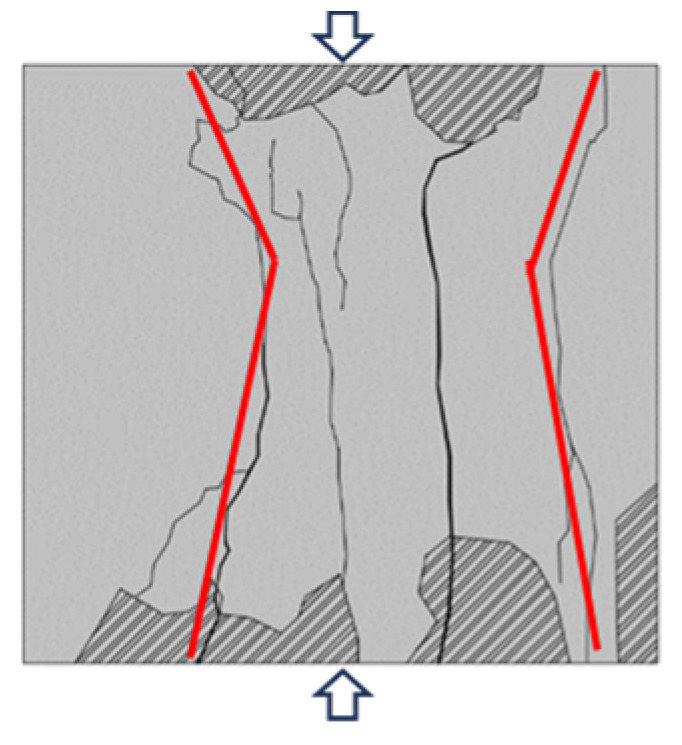

## Data Availability

The data used to support the findings of this study are included in the article.

## Supplementary Materials

The following supporting information can be downloaded at: https://www.mdpi.com/article/10.3390/ma16237503/s1, Table S1: Dynamic crack test results of back–filling concrete samples.
