# Supplementary material for: Study on Dynamic Crack Expansion and Size Effect of Back–Filling Concrete under Uniaxial Compression
Source: Materials (Basel). 2023 Dec 4;16(23):7503. doi: 10.3390/ma16237503 (PMC10707263; doi:10.3390/ma16237503)
Supplement: Supplementary file 1 [file materials-2709416-supplementary.pdf › materials-2709416-supplementary.pdf]

Table S1. Dynamic crack test results of back-filling concrete samples

Pattern	Number of cracks		F-Meature	mAp@0.5	GIoU loss
	TF	CSF			
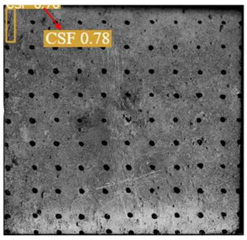	0	1	75%	71%	0.038
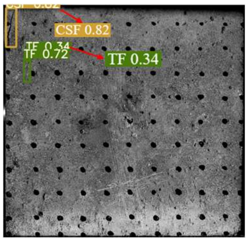	2	1			
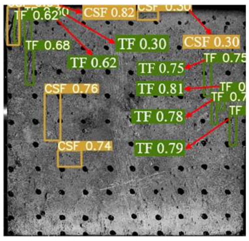	7	4			
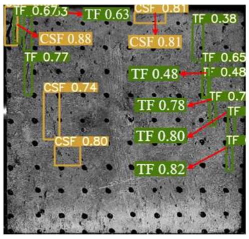	9	4			

Note: Two colors of the prediction box: green and yellow correspond to two different types of cracks: TF and CSF; The value in the prediction box is confidence.
